# Elevated TFR1 is associated with inflammatory burden and ferroptosis in ulcerative colitis

**DOI:** 10.3389/fmed.2026.1812623

**Published:** 2026-06-03

**Authors:** Xiaotong He, Jieru Ji, Lingmei Feng, Guoyu Chen, Yao Yao

**Affiliations:** Department of Gastroenterology, Shanghai Pudong New Area People's Hospital, Shanghai, China

**Keywords:** biomarker, ferroptosis, inflammation, transferrin receptor 1 (TFR1), ulcerative colitis

## Abstract

**Background:**

Ulcerative colitis (UC) is a chronic inflammatory bowel disease characterized by mucosal inflammation, oxidative stress, and iron metabolism dysregulation. Ferroptosis, an iron-dependent form of regulated cell death, may contribute to the pathogenesis of UC. Transferrin receptor 1 (TFR1), a key mediator of cellular iron uptake, links iron dysregulation to inflammation and ferroptosis; however, its clinical relevance in UC is not well defined.

**Methods:**

Serum samples were collected from 83 patients with active UC and 80 age- and sex-matched healthy controls. Disease activity was assessed using the modified Mayo score. Serum TFR1, inflammatory cytokine, coagulation indices, oxidative stress marker, and ferroptosis-related antioxidant levels were measured. Correlations and receiver operating characteristic (ROC) curve analyses were performed to evaluate the associations and diagnostic performance.

**Results:**

Serum TFR1 levels were significantly higher in patients with UC than in controls (*p* < 0.001) and increased progressively with disease severity. TFR1 showed positive correlations with the Mayo score (*r* = 0.510), inflammatory markers, including C-reactive protein (CRP; *r* = 0.350), tumor necrosis factor-alpha (TNF-α; *r* = 0.406), interleukin-1 beta (IL-1β; *r* = 0.471), and interleukin-6 (IL-6; *r* = 0.432), coagulation parameters, including platelet count (*r* = 0.225), erythrocyte sedimentation rate (ESR; *r* = 0.451), and D-dimer (*r* = 0.447), serum iron (*r* = 0.267), and lipid peroxidation (*r* = 0.315), and negative correlations with the antioxidant defenses glutathione peroxidase 4 (GPX4; *r* = −0.236) and glutathione (GSH; *r* = −0.302). ROC analysis demonstrated that serum TFR1 distinguished patients with UC from controls, with an AUC of 0.795 at a cutoff of 4.8 ng/mL.

**Conclusion:**

Elevated serum TFR1 levels are associated with UC severity, systemic inflammation, oxidative stress, and ferroptosis-related imbalance. These findings suggest that TFR1 is a potential biomarker for UC activity and highlight iron-driven ferroptosis as a promising therapeutic target, providing novel clinical insights into disease monitoring and management.

## Graphical abstract

**Figure:**
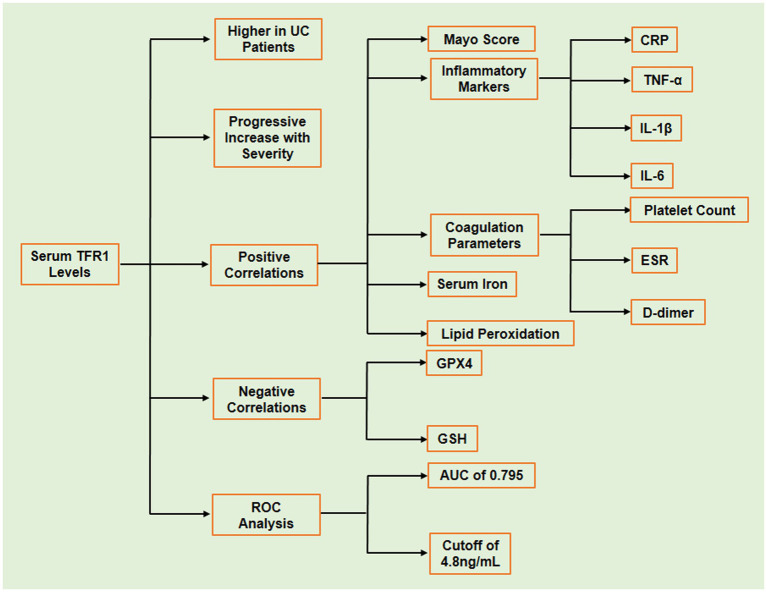
Association of serum transferrin receptor 1 (TFR1) with disease activity, inflammation, coagulation, and ferroptosis-related markers in ulcerative colitis (UC). Schematic overview of the key findings of this study. Serum TFR1 levels were significantly higher in patients with UC than in healthy controls and increased progressively with disease severity. Elevated TFR1 showed positive correlations with clinical disease activity (Mayo score), inflammatory markers (CRP, TNF-α, IL-1β, and IL-6), coagulation parameters (platelet count, ESR, and D-dimer), serum iron, and lipid peroxidation, indicating enhanced inflammatory, prothrombotic, and oxidative stress states. In contrast, TFR1 levels were negatively correlated with the ferroptosis-protective antioxidants GPX4 and GSH. Receiver operating characteristic analysis demonstrated good diagnostic performance of serum TFR1 for UC, with an area under the curve of 0.795 and an optimal cutoff value of 4.8 ng/mL.

## 1 Introduction

Ulcerative colitis (UC) is a chronic relapsing inflammatory bowel disease (IBD) that primarily affects the colonic mucosa and significantly impairs the quality of life of patients worldwide. Its incidence and prevalence are increasing globally, with a substantial healthcare burden due to frequent disease flares, hospitalizations, and an increased risk of colorectal neoplasia. Recent global epidemiological data confirm the rising prevalence and incidence of IBD in both developed and newly industrialized regions, underscoring IBD as a major global health concern (1, 2). Current evidence indicates that UC pathogenesis results from complex interactions between genetic predisposition, environmental factors, dysregulated immune responses, and impaired intestinal barrier integrity, with gut microbiota dysbiosis emerging as a key contributor to mucosal immune dysfunction (3, 4). Despite advances in immunomodulatory therapies, many patients continue to exhibit refractory disease or experience serious treatment-related adverse events, highlighting that a substantial proportion of patients are primary non-responders or lose response over time, underscoring the need for novel mechanistic insights that can inform targeted therapeutics (5).

Among the multiple drivers of UC, iron metabolism dysregulation and oxidative stress have emerged as key pathophysiological contributors. Iron is indispensable for cellular processes, including DNA synthesis, mitochondrial function, and immune cell proliferation. Iron homeostasis is tightly controlled to prevent excessive free iron catalyzing reactive oxygen species (ROS) production via Fenton chemistry, which can cause lipid peroxidation and cellular injury (6). In inflammatory conditions, such as IBD, systemic iron handling is frequently altered. Patients commonly manifest functional iron deficiency mediated by hepcidin upregulation driven by pro-inflammatory cytokines, such as interleukin-6 (IL-6), which sequesters iron within macrophages and reduces its availability for erythropoiesis, contributing to anemia of chronic disease (ACD) and iron deficiency anemia (IDA) seen in patients with UC and Crohn’s (7, 8).

Transferrin receptor 1 (TFR1), also known as CD71, is a transmembrane glycoprotein that mediates cellular iron acquisition by binding transferrin-bound iron and internalizing it into cells for metabolic use (9). In systemic iron depletion, iron regulatory proteins (IRP1 and IRP2) bind to iron-responsive elements (IREs) in the 3′-UTR of TFR1 mRNA, stabilizing transcripts and increasing receptor expression to meet cellular iron demand via the IRE/IRP network (10). Clinically, the soluble fragment of TFR1 (sTFR1), a cleaved extracellular portion found in serum, serves as a surrogate marker of iron deficiency and erythropoietic activity in disorders such as iron-deficient anemia and chronic inflammatory states, as its levels rise with increased TfR1 expression and iron demand (11).

More recently, research on UC has revealed that ferroptosis, a regulated, iron-dependent form of cell death characterized by overwhelming lipid peroxidation and reactive oxygen species, may significantly contribute to mucosal injury and is mechanistically distinct from apoptosis or necrosis (12, 13). Ferroptosis is regulated by glutathione peroxidase 4 (GPX4), glutathione (GSH), and iron availability; depletion of GPX4 or GSH leads to the lethal accumulation of lipid peroxides, especially in settings of elevated labile iron (12). Emerging preclinical studies have demonstrated that ferroptosis markers are elevated in experimental colitis models and human UC tissues, and that pharmacologic inhibition of ferroptosis, for example, via activation of the Nrf2-GPX4 axis, ameliorates colonic inflammation and promotes mucosal healing (14, 15). These findings position ferroptosis as a biomarker of tissue oxidative injury and a potential therapeutic target in UC (16).

However, despite the growing interest in ferroptosis and iron metabolism in UC, critical gaps remain regarding the clinical relevance of iron uptake pathways, particularly TFR1, in human disease. Prior studies have primarily focused on tissue-level changes in ferroptotic regulators and iron handling in animal models and human mucosal specimens; however, mechanistic insights have yet to be fully translated into accessible clinical markers (e.g., circulating biomarkers) (14, 16). While studies on UC biopsies have demonstrated ferroptosis-associated lipid peroxidation and iron dysregulation, investigations into circulating markers of iron-driven oxidative cell death in patients with active UC remain limited (17). Importantly, the relationships between circulating TFR1 levels, systemic inflammation, iron parameters, oxidative stress, and disease severity in UC have not been comprehensively evaluated, although sTFR1 has been explored as an inflammation-independent indicator of iron demand in IBD (18). Understanding this relationship is clinically relevant because it can bridge mechanistic insights into iron dysregulation with accessible biomarkers that may guide risk stratification, disease monitoring, and therapy.

In this study, we tested the hypothesis that serum TFR1 levels are elevated in patients with active UC and correlate with clinical disease activity, systemic inflammation, oxidative stress, and ferroptosis-related biomarkers, distinguishing these patients from healthy controls. By characterizing the clinical signature of TFR1 in UC, we aimed to clarify whether TFR1 serves not only as a marker of dysregulated iron metabolism but also as an integrated indicator of disease severity and ferroptotic imbalance. Our findings have the potential to refine biomarker panels for UC and highlight novel pathways for therapeutic modulation.

## 2 Methods

### 2.1 Study population

This prospective study enrolled 83 patients with UC from the Department of Gastroenterology at our hospital between January 2022 and December 2024. UC diagnosis was established according to the 2017 European Society for Clinical Nutrition and Metabolism (ESPEN) guidelines (19, 20). The inclusion criteria were as follows: (i) age ≥18 years, (ii) first diagnosis of UC, and (iii) complete clinical data. The exclusion criteria were as follows: (i) current or recent smoking or excessive alcohol consumption, (ii) other systemic inflammatory, infectious, or autoimmune diseases, (iii) family history of UC, (iv) prior colon-related surgeries, (v) hematopoietic or hepatobiliary disorders, coagulation abnormalities, or autoimmune disorders, (vi) history of malignancy, and (vii) receipt of iron supplementation, anemia-related pharmacologic therapy, or adherence to special iron-restricted or iron-fortified diets within 3 months prior to enrollment to minimize confounding effects on serum iron indices and TFR1 levels. Age- and sex-matched healthy individuals who underwent routine physical examination were included as controls. The study protocol was approved (Approval No. 2025-LW-02) by the Ethics Committee of the Shanghai Pudong New Area People’s Hospital.

### 2.2 Evaluation of disease severity

The severity of UC was assessed using the modified Mayo scoring system, which evaluates rectal bleeding, bowel movement frequency, physician assessment, and endoscopic findings. Each item is scored from 0 to 3, for a total score of 0 to 12. Scores of 0–2 indicate clinical remission, while scores above 2 indicate active disease. Scores of 3–5 indicate mild disease activity, 6–10 moderate, and 11–12 severe (21–24).

### 2.3 Data collection

We collected clinical data for each patient, including age, sex, and body mass index (BMI). Fasting venous blood samples were obtained within 24 h of admission (25). Albumin, hemoglobin, white blood cells (WBC), platelets, erythrocyte sedimentation rate (ESR), and C-reactive protein (CRP) levels were measured using an automatic biochemical analyzer. D-Dimer was assessed with an automated coagulation analyzer (26). Serum iron (Fe) levels were determined using an iron assay kit (A039-1-1, Nanjing Jiancheng Bioengineering Institute). Lipid peroxide (LPO) levels were measured using an LPO Content Assay Kit (BC5245, Solarbio) and a spectrophotometer.

### 2.4 ELISA assay

Serum biomarker concentrations were quantified in both the control subjects and patients with UC. Enzyme-linked immunosorbent assay (ELISA) kits were used for this purpose. The following kits were used: tumor necrosis factor-α (TNF-α; DTA00D, R&D Systems), interleukin 1β (IL-1β; DLB50, R&D Systems), interleukin 6 (IL-6; D6050B, R&D Systems), glutathione peroxidase 4 (GPX4; ml060706, Shanghai Enzyme-linked Biotechnology), glutathione (GSH; E-EL-0026, Elabscience), and transferrin receptor 1 (TFR1; DY2474, R&D Systems). All samples were tested in duplicate, and intra-assay CV was <8% and inter-assay CV was <10%.

### 2.5 Statistical analysis

We used IBM SPSS Statistics version 20.0 for all the statistical analyses. For normally distributed continuous variables, we reported the means and standard deviations. We compared two groups using an independent-samples *t*-test and more than two groups using a one-way ANOVA. Categorical variables are presented as frequencies and were analyzed using the chi-square test. Pearson’s correlation was used to examine the relationship between serum TFR1 levels and other continuous variables. To identify the optimal cutoff for serum TFR1 levels to distinguish patients with ulcerative colitis from healthy controls, we performed a receiver operating characteristic (ROC) curve analysis. Results were considered statistically significant if *p* was less than 0.05.

## 3 Results

### 3.1 Clinical and laboratory characteristics of study participants

The demographic and baseline characteristics of the UC and control groups are presented in Table 1. No significant differences were observed in age (*p* = 0.470), sex (*p* = 0.712), or body mass index (*p* = 0.053), indicating adequate comparability between the groups.

**Table 1:** Clinic and laboratory characteristics of study population.

Variable	Controls (*n* = 80)	Colitis (*n* = 83)	*p*-value
Age (years)	43.63 ± 8.94	42.61 ± 8.87	0.470
Sex (male, %)	43 (53.8%)	47 (56.6%)	0.712
BMI (kg/m^2^)	24.61 ± 2.57	23.78 ± 2.82	0.053
Serum albumin (g/L)	41.90 ± 3.45	36.15 ± 3.91***	<0.001
Hemoglobin	140.49 ± 16.38	126.94 ± 14.05***	<0.001
WBC (×10^9^/L)	5.68 ± 1.75	9.59 ± 3.05***	<0.001
Platelet (×10^9^/L)	217.41 ± 45.88	253.74 ± 54.62***	<0.001
ESR (mm/h)	6.68 ± 2.01	16.55 ± 5.76***	<0.001
D-Dimer (μg/L)	216.08 ± 33.43	523.31 ± 117.53***	<0.001
CRP (μg/mL)	2.80 ± 0.58	17.15 ± 4.38***	<0.001
TNF-α (ng/mL)	48.00 ± 6.23	92.21 ± 23.42***	<0.001
IL-1β (ng/mL)	2.28 ± 0.42	4.61 ± 0.99***	<0.001
IL-6 (ng/mL)	3.71 ± 0.68	18.13 ± 5.77***	<0.001
Iron (mg/L)	27.37 ± 3.83	39.19 ± 5.89***	<0.001
LPO (nmol/mL)	8.57 ± 1.27	11.89 ± 2.08***	<0.001
GPX4 (ng/mL)	17.56 ± 3.40	14.03 ± 2.82***	<0.001
GSH (nmol/mL)	9.72 ± 1.57	7.05 ± 1.37***	<0.001
TFR1 (ng/mL)	4.27 ± 0.71	5.29 ± 0.99***	<0.001

*T*-test is applied to compare the differences between two groups. *p < 0.05, **p < 0.01, ***p < 0.001 vs controls.

BMI, body mass index; WBC, white blood cells; ESR, erythrocyte sedimentation rate; CRP, C-reactive protein; TNF-α, tumor necrosis factor-α; IL-1β, interleukin 1β; IL-6, interleukin 6; LPO, lipid peroxide; GPX4, glutathione peroxidase 4; GSH, glutathione; TFR1, transferrin receptor 1.

Patients with UC exhibited markedly altered laboratory profiles compared to those of the controls, reflecting systemic inflammation, oxidative stress, and disrupted iron metabolism. Albumin and hemoglobin were reduced (all *p* < 0.001), whereas WBC count, platelet count, ESR, D-dimer, and CRP were elevated (all *p* < 0.001). Proinflammatory cytokines (TNF-α, IL-1β, IL-6) and oxidative stress markers (serum iron, LPO) were significantly higher in patients with UC, whereas antioxidant defenses (GPX4, GSH) were lower (all *p* < 0.01). Notably, serum TFR1 levels were higher in patients with UC than in controls (*p* < 0.001), suggesting enhanced iron uptake and potential ferroptotic signaling.

### 3.2 Clinical and biochemical changes across disease severity

Patients were stratified by disease activity into mild, moderate, and severe groups (Table 2). Demographic variables remained comparable across the severity groups (age: *p* = 0.542; sex: *p* = 0.963; BMI: *p* = 0.121). Progressive and significant increases were observed in the WBC count (*p* < 0.001), platelet count (*p* = 0.031), ESR, D-dimer, CRP, TNF-α, IL-1β, and IL-6 (all *p* < 0.001) with increasing disease severity. Oxidative and iron-related markers, including serum iron (*p* = 0.002) and LPO (*p* < 0.001), increased progressively, whereas GPX4 (*p* = 0.006) and GSH (*p* = 0.011) decreased. Importantly, TFR1 levels increased stepwise with disease severity (*p* < 0.001), highlighting its association with escalating clinical and biochemical indices of inflammation and oxidative stress.

**Table 2:** Clinic and laboratory characteristics of colitis patients with different severity.

Variable	Mild group (*n* = 34)	Moderate group (*n* = 27)	Severe group (*n* = 22)	*p*-value
Age (years)	43.88 ± 7.24	42.04 ± 10.23	41.36 ± 9.52	0.542
Sex (male, %)	19 (55.9%)	15 (55.6%)	13 (56.6%)	0.963
BMI (kg/m^2^)	24.36 ± 3.07	23.86 ± 2.58	22.79 ± 2.53*	0.121
Serum albumin (g/L)	37.27 ± 3.77	35.68 ± 3.72	34.99 ± 4.05*	0.075
Hemoglobin	129.75 ± 15.54	125.68 ± 13.69	124.15 ± 11.67	0.297
WBC (×10^9^/L)	8.49 ± 2.68	9.30 ± 2.62	11.65 ± 3.17***^##^	<0.001
Platelet (×10^9^/L)	236.14 ± 50.33	259.62 ± 51.79	273.74 ± 58.16*	0.031
ESR (mm/h)	11.51 ± 2.35	17.46 ± 3.07***	23.23 ± 4.50***^###^	<0.001
D-Dimer (μg/L)	429.54 ± 64.42	533.63 ± 74.61***	655.55 ± 88.47***^###^	<0.001
CRP (μg/mL)	13.78 ± 1.93	16.97 ± 2.77***	22.57 ± 3.23***^###^	<0.001
TNF-α (ng/mL)	73.70 ± 9.54	91.62 ± 13.20***	121.55 ± 18.07***^###^	<0.001
IL-1β (ng/mL)	3.79 ± 0.64	4.87 ± 0.69***	5.54 ± 0.71***^###^	<0.001
IL-6 (ng/mL)	12.98 ± 1.55	18.72 ± 3.37***	25.37 ± 3.69***^###^	<0.001
Iron (mg/L)	36.57 ± 5.68	40.29 ± 4.56*	41.88 ± 6.24***	0.002
LPO (nmol/mL)	10.68 ± 1.65	12.49 ± 1.84***	13.02 ± 2.05***	<0.001
GPX4 (ng/mL)	15.18 ± 3.08	13.45 ± 2.06*	12.99 ± 2.69**	0.006
GSH (nmol/mL)	7.55 ± 1.35	6.90 ± 1.20	6.47 ± 1.39**	0.011
TFR1 (ng/mL)	4.71 ± 0.68	5.37 ± 0.90**	6.08 ± 0.95***^##^	<0.001

ANOVA is applied to compare the differences between three groups. *p < 0.05, **p < 0.01, *p < 0.001 vs mild group; #*p* < 0.05, ##*p* < 0.01, ###*p* < 0.001 vs moderate group.

BMI, body mass index; WBC, white blood cells; ESR, erythrocyte sedimentation rate; CRP, C-reactive protein; TNF-α, tumor necrosis factor-α; IL-1β, interleukin 1β; IL-6, interleukin 6; LPO, lipid peroxide; GPX4, glutathione peroxidase 4; GSH, glutathione; TFR1, transferrin receptor 1.

### 3.3 Serum TFR1 levels and disease activity

**Figure 1:**
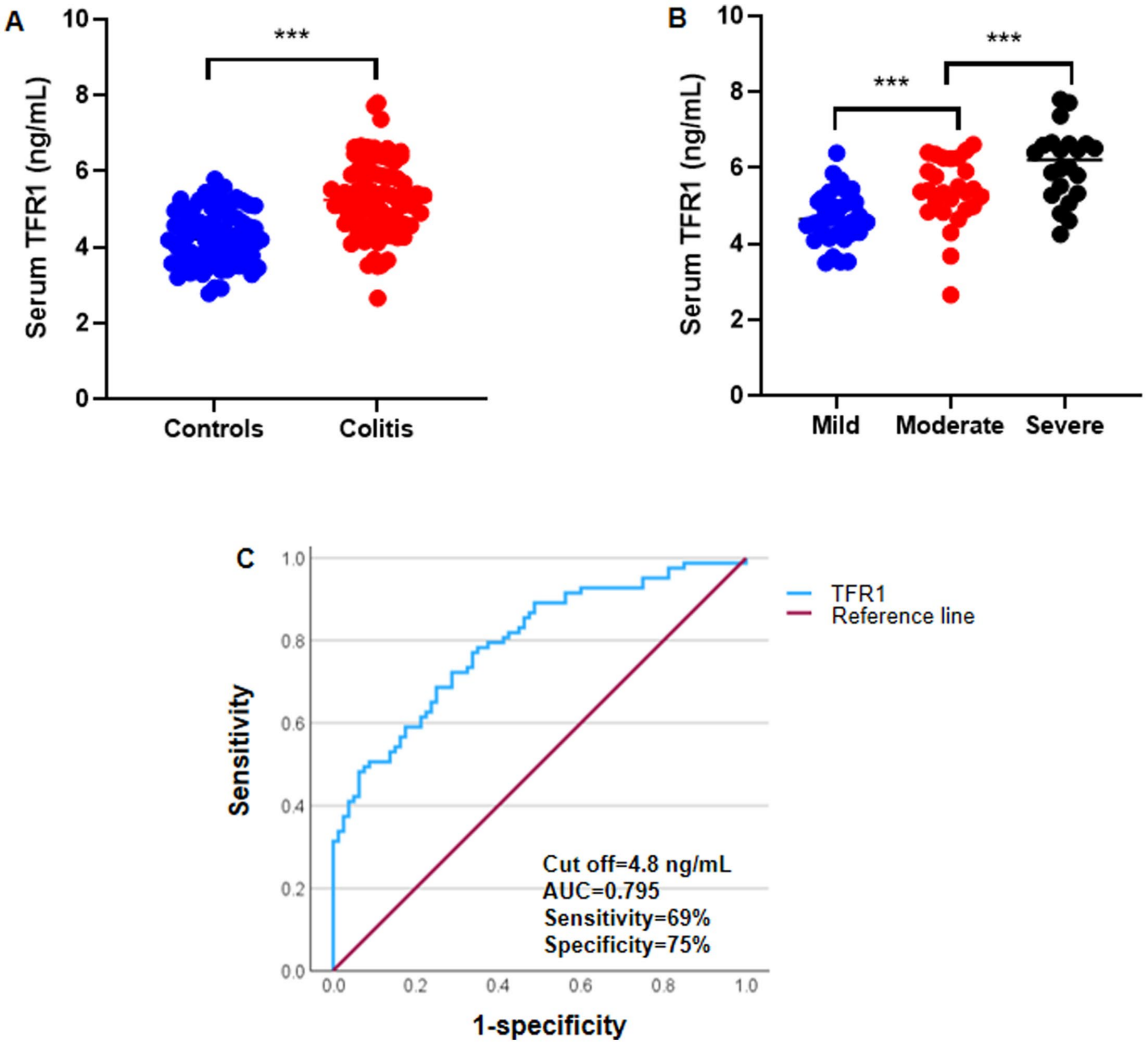
Serum transferrin receptor 1 (TFR1) levels in patients with ulcerative colitis (UC) and their diagnostic performance. **(A)** Comparison of serum TFR1 levels between healthy controls (blue) and patients with UC (red). **(B)** Serum TFR1 levels stratified by UC severity (mild, moderate, severe). Data are shown as individual points; ***p < 0.001. The *t*-test and ANOVA were used to compare differences between two and three groups, respectively. **(C)** Receiver operating characteristic (ROC) curve analysis of serum TFR1 levels for discriminating UC patients from healthy controls. The area under the curve (AUC) was 0.795, with a cutoff value of 4.8 ng/mL, sensitivity of 69%, and specificity of 75%. TFR1, transferrin receptor 1; UC, ulcerative colitis.

Serum TFR1 levels were significantly elevated in patients with UC compared to those in healthy controls (*p* < 0.001; Figure 1A). Stratification by disease severity revealed a progressive elevation in the mild, moderate, and severe UC groups (Figure 1B). ROC curve analysis demonstrated that TFR1 distinguished UC patients from controls with an AUC of 0.795 and an optimal cutoff of 4.8 ng/mL balancing sensitivity and specificity (Figure 1C).

### 3.4 Diagnostic performance of serum TFR1 in ulcerative colitis

**Figure 2:**
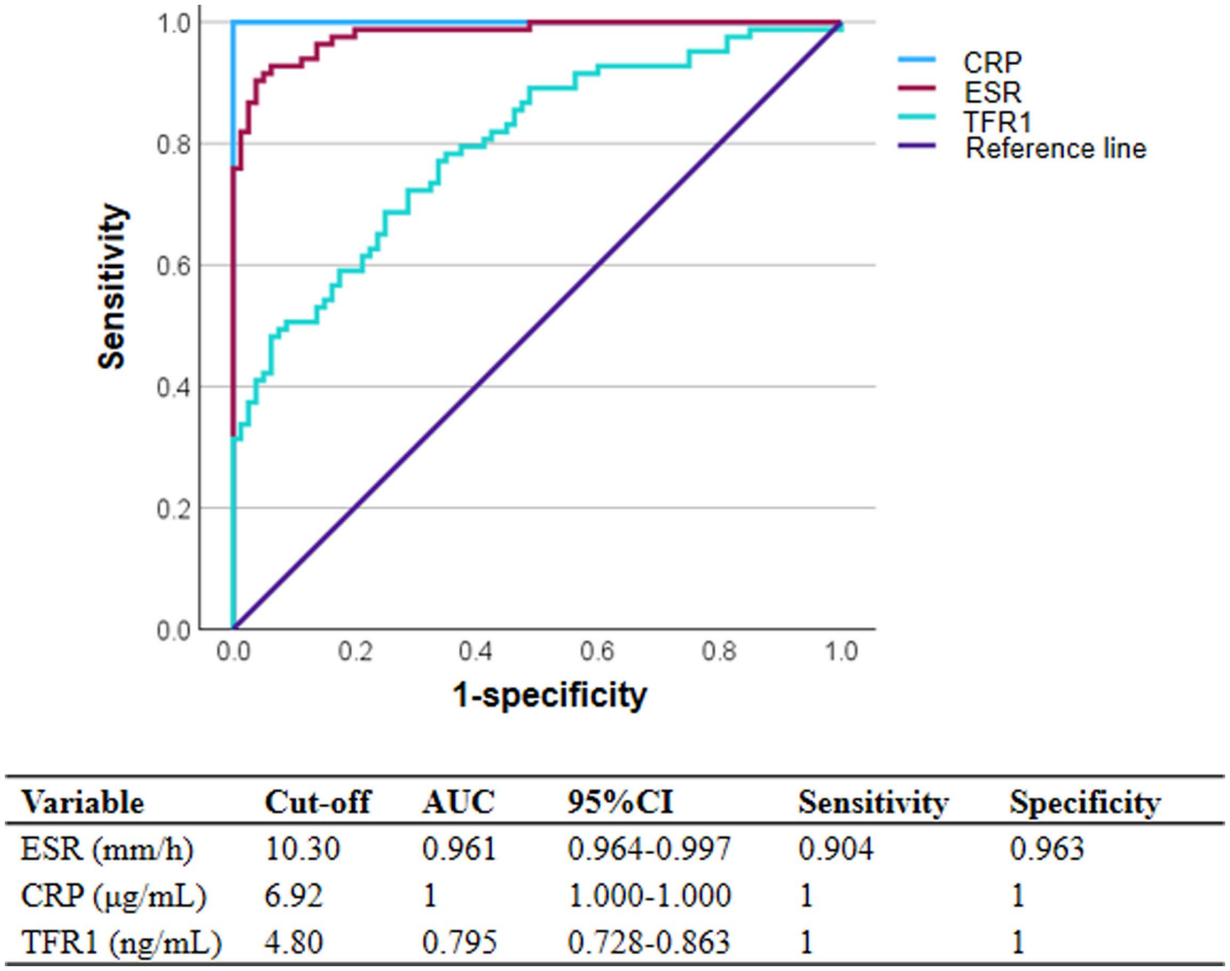
Receiver operating characteristic (ROC) curve analysis comparing the diagnostic performance of serum transferrin receptor 1 (TFR1), C-reactive protein (CRP), and erythrocyte sedimentation rate (ESR) in distinguishing ulcerative colitis patients from healthy controls. The area under the curve (AUC), optimal cutoff values, sensitivity, and specificity for each marker are shown in the accompanying table. Serum TFR1 demonstrated an AUC of 0.795 (cutoff 4.8 ng/mL) with 100% sensitivity and specificity, indicating its potential utility as a complementary biomarker for assessing disease activity.

ROC curve analysis was performed to evaluate the discriminative ability of serum TFR1 compared with traditional inflammatory markers, ESR and CRP, in patients with UC (Figure 2). Serum TFR1 effectively distinguished patients with UC from healthy controls, with an AUC of 0.795 (95% CI: 0.728–0.863) at a cutoff value of 4.8 ng/mL, achieving 100% sensitivity and specificity. For comparison, CRP demonstrated perfect discrimination (AUC = 1.0), while ESR also showed high diagnostic accuracy (AUC = 0.961, 95% CI: 0.964–0.997; sensitivity 90.4%, specificity 96.3%). These findings indicate that serum TFR1 has strong potential as a complementary biomarker for UC diagnosis and disease activity monitoring.

### 3.5 Correlation of TFR1 with inflammatory and coagulation markers

**Figure 3:**
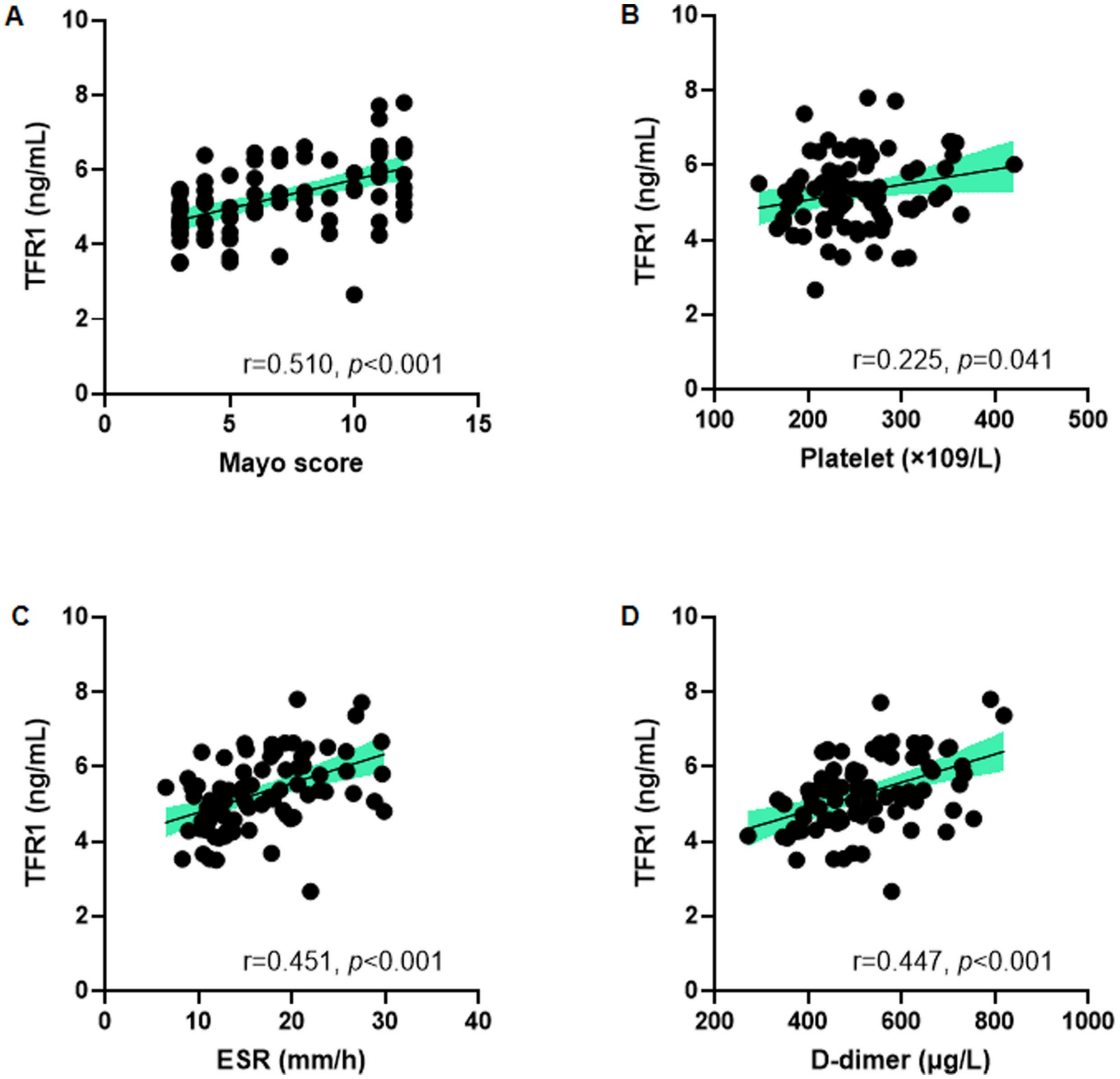
Correlation of serum transferrin receptor 1 (TFR1) with clinical and laboratory parameters in ulcerative colitis (UC) patients. Scatter plots depicting the relationships between serum TFR1 levels and **(A)** modified Mayo score, **(B)** platelet count, **(C)** erythrocyte sedimentation rate (ESR), and **(D)** D-dimer levels. Spearman correlation coefficients (*r*) and *p*-values are indicated in each panel. TFR1 levels were positively correlated with disease activity and inflammatory/coagulation markers in patients with UC.

**Figure 4:**
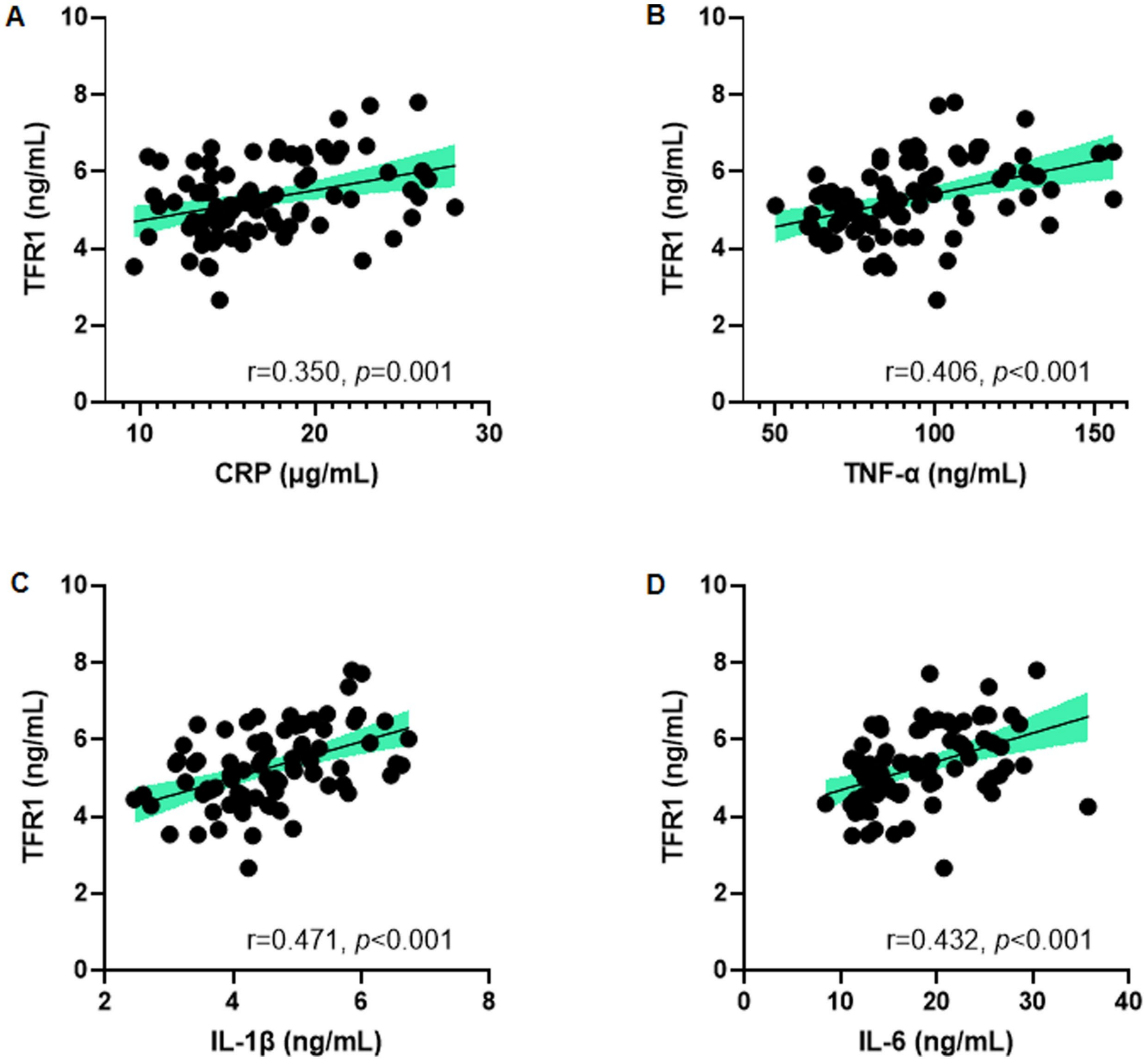
Correlation between serum TFR1 levels and inflammatory indicators of UC patients. Serum TFR1 is positively correlated with **(A)** CRP, **(B)** TNF-α, **(C)** IL-1β, and **(D)** IL-6. CRP, C-reactive protein; TNF-α, tumor necrosis factor-α; IL-1β, interleukin 1β; IL-6, interleukin 6.

Pearson’s correlation analysis revealed strong positive correlations between serum TFR1 and disease activity (Mayo score, *r* = 0.510; *p* < 0.001), platelet count (*r* = 0.225; *p* = 0.041), ESR (*r* = 0.451; *p* < 0.001), D-dimer (*r* = 0.447; *p* < 0.001), CRP (*r* = 0.350; *p* = 0.001), TNF-α (*r* = 0.406; *p* < 0.001), IL-1β (*r* = 0.471; *p* < 0.001), and IL-6 (*r* = 0.432; *p* < 0.001) (Figures 3, 4). These results indicate that TFR1 reflects the systemic inflammatory burden and coagulation activation in UC.

### 3.6 Association with ferroptosis-related biomarkers

**Figure 5:**
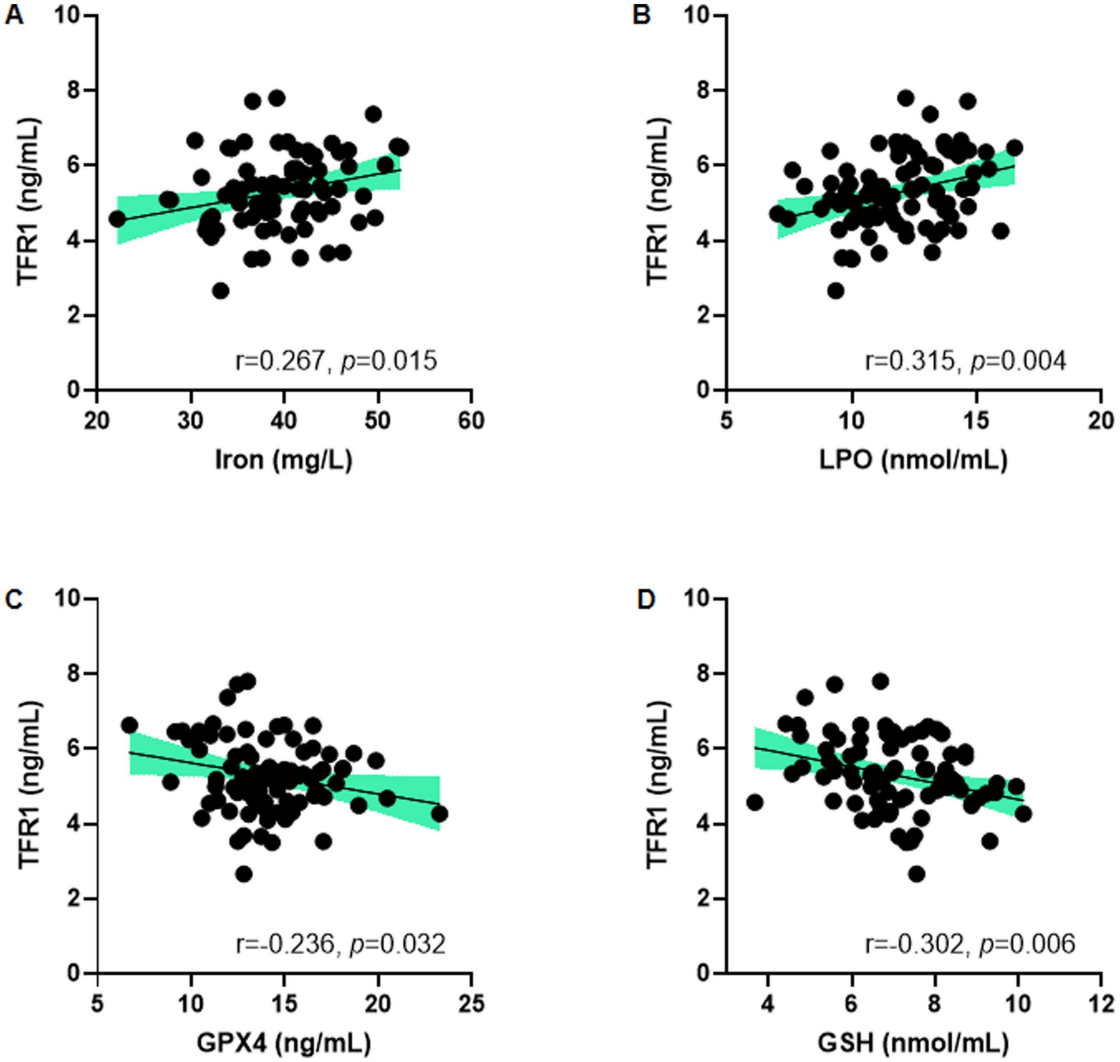
Associations between serum TFR1 and iron, oxidative stress, and antioxidant markers in ulcerative colitis (UC) patients. Scatter plots depicting the correlations between serum transferrin receptor 1 (TFR1) and key biochemical parameters in patients with ulcerative colitis (UC). **(A,B)** TFR1 positively correlated with serum iron and lipid peroxidation (LPO) levels. **(C,D)** TFR1 negatively correlated with glutathione peroxidase 4 (GPX4) concentrations and glutathione (GSH) levels. These results indicate that elevated TFR1 is associated with increased iron burden and oxidative stress, along with reduced antioxidant defenses, supporting its potential involvement in ferroptosis-related processes in UC.

Serum TFR1 levels were positively associated with serum iron (*r* = 0.267; *p* = 0.015) and LPO (*r* = 0.315; *p* = 0.004) and negatively associated with antioxidant markers GPX4 (*r* = −0.236; *p* = 0.032) and GSH (*r* = −0.302; *p* = 0.006) (Figure 5). These correlations suggest that elevated TFR1 levels may indicate increased iron-driven oxidative stress and ferroptotic activity.

## 4 Discussion

In this study, we demonstrated that serum TFR1 levels were markedly elevated in patients with UC compared to healthy controls and increased progressively with disease severity. Elevated TFR1 levels were positively correlated with established inflammatory markers (CRP, ESR, and proinflammatory cytokines), coagulation indices (platelet count and D-dimer), and ferroptosis-related biomarkers (serum iron and lipid peroxidation), while being inversely correlated with key antioxidant defenses (GPX4 and GSH). These results support a multifaceted role for TFR1 as a potential biomarker of systemic inflammation, iron dysregulation, oxidative stress, and ferroptotic propensity in UC.

Our results are consistent with an expanding body of evidence identifying ferroptosis as a critical contributor to UC pathogenesis. Ferroptosis is a regulated form of cell death characterized by iron-dependent lipid peroxidation and impaired antioxidant defense. It has emerged as a mechanistic link between iron metabolism, oxidative injury, immune activation, and intestinal barrier dysfunction in inflammatory bowel disease. Recent comprehensive reviews have emphasized that excessive iron accumulation and depletion of antioxidant systems promote lipid peroxidation–mediated mucosal injury in UC and Crohn’s disease (17). In parallel, experimental and translational studies have demonstrated that iron overload, reactive oxygen species accumulation, and lipid peroxidation synergistically disrupt epithelial integrity and amplify intestinal inflammation, positioning ferroptosis as a central pathological mechanism in UC (16).

These mechanistic insights were strongly supported by our correlation analyses. TFR1 is the principal cell-surface receptor that mediates the uptake of transferrin-bound iron, and its upregulation increases intracellular labile iron pools, thereby facilitating Fenton chemistry-driven reactive oxygen species generation. This process promotes lipid peroxidation, a hallmark of ferroptosis, particularly when antioxidant defenses, such as the system Xc^−^-GSH-GPX4 axis, are compromised. In our cohort, higher TFR1 levels were associated with increased serum iron and lipid peroxidation, alongside reduced GPX4 and GSH levels, consistent with a ferroptosis-prone biochemical milieu. Ferroptosis is mechanistically distinct from apoptosis and necroptosis because of its strict dependence on iron overload and lethal lipid peroxide accumulation. Experimental models of colitis have shown that excess iron, GPX4 inactivation, and glutathione depletion exacerbate epithelial injury and barrier dysfunction (14, 27–29). These observations provide a strong biological framework for the clinical associations identified in our study.

Beyond its mechanistic relevance, ferroptosis has gained increasing attention as a potential therapeutic target for UC. Recent experimental studies have demonstrated that pharmacological or molecular inhibition of ferroptosis can attenuate intestinal inflammation and tissue injury. Activation of hypoxia-inducible factor-1α suppresses ferroptosis by upregulating GPX4 expression, thereby alleviating colitis severity in both UC tissues and animal models (14). Similarly, modulation of the farnesoid X receptor has been reported to inhibit ferroptotic pathways and reduce intestinal inflammation, further supporting the therapeutic relevance of targeting iron-dependent lipid peroxidation in UC (30). Within this context, TFR1 may serve not only as a biomarker reflecting ferroptotic activation but also as a potential surrogate indicator of the therapeutic modulation of iron-driven oxidative stress.

The clinical relevance of our findings is underscored by the strong correlation between serum TFR1 levels and disease activity, as assessed using the Mayo score. While conventional inflammatory markers such as CRP and ESR remain indispensable in clinical practice, they primarily reflect systemic inflammation and provide limited insight into iron metabolism–related oxidative injury or regulated cell death pathways. Emerging evidence suggests that ferroptosis contributes to intestinal inflammation through iron overload, lipid peroxidation, and impaired antioxidant defense,pathological processes that are not adequately captured by traditional inflammatory indices (29, 31). In this regard, TFR1, a key regulator of transferrin-mediated iron uptake, may offer complementary mechanistic information reflecting iron-dependent oxidative vulnerability and ferroptotic propensity in UC (32, 33). Clinically, the incorporation of ferroptosis-related biomarkers, such as TFR1, into diagnostic or monitoring strategies could improve risk stratification and aid in identifying patients with heightened oxidative and iron-driven injury, particularly those with iron dysregulation or treatment-refractory disease. Longitudinal assessment of serum TFR1 levels before and after therapeutic intervention may further clarify whether specific treatments modulate ferroptosis-related pathways, providing insights beyond those of conventional inflammatory markers (34, 35).

Additionally, the observed associations between TFR1 and coagulation-related indices suggest a potential link between iron-driven oxidative stress and the prothrombotic state commonly observed in patients with active UC. Iron overload and lipid peroxidation have been implicated in endothelial dysfunction, platelet activation, and microvascular injury, which may contribute to thrombo-inflammatory complications in IBD (14, 36). Consistent with this concept, iron metabolism markers, such as soluble transferrin receptor, are being explored as inflammation-independent indicators of iron demand in UC, highlighting the growing recognition of iron handling in disease monitoring (18). Furthermore, increased mucosal expression of TFR1 in inflamed IBD tissue supports the involvement of TFR1-related pathways in the local inflammatory microenvironment (37). Given the elevated risk of venous thromboembolism and systemic hypercoagulability in active UC, understanding how iron dysregulation and oxidative stress intersect with coagulation pathways is of clinical importance (38).

Despite the strengths of our study, its cross-sectional design precludes causal inference. Whether elevated TFR1 actively drives disease progression through ferroptotic mechanisms or represents a downstream consequence of inflammatory signaling remains unclear. Longitudinal studies integrating circulating biomarkers with tissue-level ferroptosis assays are essential for clarifying temporal relationships and establishing causality. Nonetheless, our findings provide clinical evidence linking TFR1 to inflammation, iron dysregulation, oxidative stress, and ferroptosis in UC, supporting its potential role as a biomarker and translational target for future research.

## 5 Limitations

This study has several limitations. First, its cross-sectional and observational design precludes causal inference; thus, the associations between serum TFR1 levels, disease activity, inflammatory burden, and ferroptosis-related markers should be interpreted as correlative rather than mechanistic. Second, all analyses were based on circulating serum markers without direct tissue-level validation of the results. Colonic biopsy-based assessments, such as immunohistochemistry, western blotting, or molecular analyses of TFR1 expression and ferroptosis-related pathways, were not performed. Consequently, serum TFR1 may not fully reflect mucosal or epithelial iron handling, local ferroptotic activity, or intestinal inflammation. Third, although patients with recent iron supplementation, anemia-related therapy, or special iron-modifying diets were excluded, residual confounding from unmeasured dietary iron intake, prior anemia management, or subclinical iron deficiency cannot be entirely excluded and may have influenced the serum iron indices and TFR1 levels. Finally, direct mechanistic validation of ferroptosis was not conducted; no tissue-based assays, ultrastructural mitochondrial evaluations, pharmacological inhibitors, or cell-based experiments targeting TFR1 were performed. Inferences regarding ferroptosis were drawn solely from surrogate circulating markers, including serum iron, lipid peroxidation, and antioxidant depletion (GPX4 and glutathione), which do not provide definitive evidence of ferroptotic cell death *in vivo*. Future longitudinal and mechanistic studies using intestinal biopsies, ferroptosis-specific molecular assays, detailed dietary assessments, and functional modulation of TFR1 are warranted to confirm the mucosal relevance and evaluate the therapeutic significance of ulcerative colitis.

## 6 Conclusion

In conclusion, this study demonstrated that serum TFR1 levels were significantly elevated in patients with UC and were closely associated with disease activity, systemic inflammation, oxidative stress, and ferroptosis-related imbalance. These findings support the utility of TFR1 as a clinically relevant biomarker of inflammatory burden and altered iron metabolism in UC. However, given the correlative nature of this study and the absence of mechanistic validation, TFR1 should be interpreted as an indicator of ferroptosis susceptibility rather than a confirmed causal driver of disease pathogenesis. Further mechanistic investigations, including cell-based and *in vivo* studies targeting TFR1 and ferroptotic pathways, are required to determine whether the modulation of iron uptake or ferroptosis represents a viable therapeutic strategy for ulcerative colitis.

## Data Availability

The datasets presented in this study can be found in online repositories. The names of the repository/repositories and accession number(s) can be found in the article/Supplementary material.
