# Supplementary material for: Elevated TFR1 is associated with inflammatory burden and ferroptosis in ulcerative colitis
Source: Front Med (Lausanne). 2026 Jun 3;13:1812623. doi: 10.3389/fmed.2026.1812623 (PMC13272019; doi:10.3389/fmed.2026.1812623)
Supplement: Supplementary file 1 [file Table_1.DOC]

**Table 1.** Clinic and laboratory characteristics of study population

| **Variable** | **Controls**  **(n=80)** | **Colitis**  **(n=83)** | ***p*-value** |
| --- | --- | --- | --- |
| Age (years) | 43.63±8.94 | 42.61±8.87 | 0.470 |
| Sex (male, %) | 43 (53.8%) | 47 (56.6%) | 0.712 |
| BMI (kg/m^2^) | 24.61±2.57 | 23.78±2.82 | 0.053 |
| Serum albumin (g/L) | 41.90±3.45 | 36.15±3.91*** | <0.001 |
| Hemoglobin | 140.49±16.38 | 126.94±14.05*** | <0.001 |
| WBC (×10^9^/L) | 5.68±1.75 | 9.59±3.05*** | <0.001 |
| Platelet (×10^9^/L) | 217.41±45.88 | 253.74±54.62*** | <0.001 |
| ESR (mm/h) | 6.68±2.01 | 16.55±5.76*** | <0.001 |
| D-Dimer (μg/L) | 216.08±33.43 | 523.31±117.53*** | <0.001 |
| CRP (μg/mL) | 2.80±0.58 | 17.15±4.38*** | <0.001 |
| TNF-α (ng/mL) | 48.00±6.23 | 92.21±23.42*** | <0.001 |
| IL-1β (ng/mL) | 2.28±0.42 | 4.61±0.99*** | <0.001 |
| IL-6 (ng/mL) | 3.71±0.68 | 18.13±5.77*** | <0.001 |
| Iron (mg/L) | 27.37±3.83 | 39.19±5.89*** | <0.001 |
| LPO (nmol/mL) | 8.57±1.27 | 11.89±2.08*** | <0.001 |
| GPX4 (ng/mL) | 17.56±3.40 | 14.03±2.82*** | <0.001 |
| GSH (nmol/mL) | 9.72±1.57 | 7.05±1.37*** | <0.001 |
| TFR1 (ng/mL) | 4.27±0.71 | 5.29±0.99*** | <0.001 |

T-test is applied to compare the differences between two groups. *P<0.05, **P<0.01, ***P<0.001 vs Controls.

**Abbreviations:** BMI, body mass index; WBC, white blood cells; ESR, erythrocyte sedimentation rate; CRP, C-reactive protein; TNF-α, tumor necrosis factor-α; IL-1β, interleukin 1β; IL-6, interleukin 6; LPO, lipid peroxide; GPX4, glutathione peroxidase 4; GSH, glutathione; TFR1, transferrin receptor 1.
