# Supplementary material for: Elevated TFR1 is associated with inflammatory burden and ferroptosis in ulcerative colitis
Source: Front Med (Lausanne). 2026 Jun 3;13:1812623. doi: 10.3389/fmed.2026.1812623 (PMC13272019; doi:10.3389/fmed.2026.1812623)
Supplement: Supplementary file 2 [file Table_2.DOC]

**Table 2.** Clinic and laboratory characteristics of colitis patients with different severity

| **Variable** | **Mild group**  **(n=34)** | **Moderate group**  **(n=27)** | **Severe group**  **(n=22)** | ***p*-value** |
| --- | --- | --- | --- | --- |
| Age (years) | 43.88±7.24 | 42.04±10.23 | 41.36±9.52 | 0.542 |
| Sex (male, %) | 19 (55.9%) | 15 (55.6%) | 13 (56.6%) | 0.963 |
| BMI (kg/m^2^) | 24.36±3.07 | 23.86±2.58 | 22.79±2.53* | 0.121 |
| Serum albumin (g/L) | 37.27±3.77 | 35.68±3.72 | 34.99±4.05* | 0.075 |
| Hemoglobin | 129.75±15.54 | 125.68±13.69 | 124.15±11.67 | 0.297 |
| WBC (×10^9^/L) | 8.49±2.68 | 9.30±2.62 | 11.65±3.17***^##^ | <0.001 |
| Platelet (×10^9^/L) | 236.14±50.33 | 259.62±51.79 | 273.74±58.16* | 0.031 |
| ESR (mm/h) | 11.51±2.35 | 17.46±3.07*** | 23.23±4.50***^###^ | <0.001 |
| D-Dimer (μg/L) | 429.54±64.42 | 533.63±74.61*** | 655.55±88.47***^###^ | <0.001 |
| CRP (μg/mL) | 13.78±1.93 | 16.97±2.77*** | 22.57±3.23***^###^ | <0.001 |
| TNF-α (ng/mL) | 73.70±9.54 | 91.62±13.20*** | 121.55±18.07***^###^ | <0.001 |
| IL-1β (ng/mL) | 3.79±0.64 | 4.87±0.69*** | 5.54±0.71***^###^ | <0.001 |
| IL-6 (ng/mL) | 12.98±1.55 | 18.72±3.37*** | 25.37±3.69***^###^ | <0.001 |
| Iron (mg/L) | 36.57±5.68 | 40.29±4.56* | 41.88±6.24*** | 0.002 |
| LPO (nmol/mL) | 10.68±1.65 | 12.49±1.84*** | 13.02±2.05*** | <0.001 |
| GPX4 (ng/mL) | 15.18±3.08 | 13.45±2.06* | 12.99±2.69** | 0.006 |
| GSH (nmol/mL) | 7.55±1.35 | 6.90±1.20 | 6.47±1.39** | 0.011 |
| TFR1 (ng/mL) | 4.71±0.68 | 5.37±0.90** | 6.08±0.95***^##^ | <0.001 |

ANOVA is applied to compare the differences between three groups. *P<0.05, **P<0.01, *P<0.001 vs Mild group; #P<0.05, ##P<0.01, ###P<0.001 vs moderate group.

**Abbreviations:** BMI, body mass index; WBC, white blood cells; ESR, erythrocyte sedimentation rate; CRP, C-reactive protein; TNF-α, tumor necrosis factor-α; IL-1β, interleukin 1β; IL-6, interleukin 6; LPO, lipid peroxide; GPX4, glutathione peroxidase 4; GSH, glutathione; TFR1, transferrin receptor 1.
